# The role of working memory in children's ability for prosodic discrimination

**DOI:** 10.1371/journal.pone.0229857

**Published:** 2020-03-09

**Authors:** Arthur Stepanov, Karmen Brina Kodrič, Penka Stateva

**Affiliations:** Center for Cognitive Science of Language, University of Nova Gorica, Nova Gorica, Slovenia; CNRS - Université d'Aix-Marseille, FRANCE

## Abstract

Previous research established that young children are sensitive to prosodic cues discriminating between syntactic structures of otherwise similarly sounding sentences in a language unknown to them. In this study, we explore the role of working memory that children might deploy for the purpose of the sentence-level prosodic discrimination. Nine-year old Slovenian monolingual and bilingual children (N = 70) were tested on a same-different prosodic discrimination task in a language unknown to them (French) and on the working memory measures in the form of forward and backward digit span and non-word repetition tasks. The results suggest that both the storage and processing components of the working memory are involved in the prosodic discrimination task.

## Introduction

Prosody or sentence-level intonational contour plays a major role in people's language comprehension ability providing acoustic cues for identifying syntactic phrases or constituents. The underlying assumption is that there is a sufficiently close match between prosodic and syntactic constituency in the world languages [1–2]. Development of the prosodic ability at large is a key factor in children mastering the syntax of their native language [3–4], and of key importance in learning of the syntax of a second or foreign language [5–7].

Evidence suggests that infants are sensitive to phrasal prosody in their mother tongue already prenatally and are able to recognize progressively smaller prosodic chunks starting at 4 months old onwards (cf. [8–10], among others). Bilingualism and music training are known to sharpen perceptual ability to sounds [11–13]. This also includes better success in the prosodic domain, such as easier recognition and discrimination of prosodic patterns as well as rhythm in language or music [14–19]. Connections between children's prosodic ability and syntactic processing were also explored. [20] tested 3,5–4,5 years old French-speaking children using sentential preambles culminating in a noun/verb homophone. Children in that study successfully exploited prosodic information in the stimuli to assign the appropriate syntactic category to the target word. Even younger, 18-month old children were shown to use sentential prosody to facilitate word learning [21]. More recently, [22] found that Slovenian-speaking elementary school children make use of a number of prosodic cues to successfully differentiate pairs of short syntactically well-formed sentences in a language unknown to them. Sentences in the pairs only differed in intonational contour. That study compared prosodic discrimination in monolingual and bilingual children as well as children who received several years of musical training. The latter two groups performed better than monolingual controls indicating a greater perceptual sensitivity associated with each type of experience. However, even though it is becoming evident that children's remarkable prosodic sensitivity may be affected by linguistic (e.g. bilingualism) as well as non-linguistic (e.g. music) factors, the above studies largely leave open the issue concerning possible source(s) of this ability. As noted above, prosodic 'bootstrapping' is essential for acquiring the syntactic rules of native as well as second language(s). Therefore, clarifying the nature of the relevant cognitive mechanisms and their role in prosodic discrimination at the sentence level is of significant importance in the general context of the language acquisition task.

One possibility is that comparing two nearly identical sentences on the basis of their prosodic signatures involves working memory, in particular, phonological short-term memory (p-STM) including a phonological storage and a subvocal rehearsal process [23]. p-STM is associated with many aspects of speech perception including greater success in learning foreign vocabulary and grammar from novel input (e.g. [24–25]), and acquisition of cross-linguistic phonological regularities [26]. In the context of a prosodic discrimination task comparing the input from two similarly sounding sentences, both sentences need to be actively maintained in the phonological buffer. Better success in discrimination can thus directly or indirectly be associated with a greater p-STM capacity in the general context of speech perception (cf. also [27]).

Depending on a particular theory behind same/different decision making, it is conceivable that a processing component of the working memory is engaged in this process as well (cf. [23], [28]). One type of such theories, dubbed 'accumulator' models, is based on the idea that (possibly noisy) evidence in the form of different features or dimensions is sampled from the stimuli and accumulated over time, terminating at a threshold associated with either a "same" or "different" response (see [29] for review). Accumulation takes place in a particular type of serial or parallel information-processing architecture which may or may not involve different schemes for producing each of the two response types [30–32]. This also implies segmentation of the input as a key component of the real time analysis based, as in our case, on prosodic cues (e.g. [33–34]), as well as a possible chunking process (see Discussion). In a prosodic discrimination task, segmented chunks of prosodic/syntactic structure of each sentence in a pair may conceivably be subjected to this kind of evidence accumulating and resolution. Therefore, processing considerations could be applicable as in any information-processing system affecting accuracy in this task. Notably, both the temporary storage and processing components of the working memory are deployed in regular sentence processing (see [35–38], among others).

The enhanced performance on the sentence discrimination task manifested by bilingual and musically trained individuals also suggests a non-trivial role of the working memory and its processing component in particular. Both population groups perform better at executive control measures than respective controls [39–42]. Bilingual experience was argued to enhance working memory-related attentional processes, if only under specific experimental conditions (for review, see [43]). Similarly, musical training was shown to enhance verbal memory for spoken words [41].

The present study explores the role of both phonological storage and processing components of the working memory for the purpose of prosodic discrimination of natural language sentences in nine-year old children. Our interest in this particular age group is two-fold. First, short-term memory at large is subject to a developmental trajectory, reaching a developmental plateau by around 8 years [44], while central executive and phonological loop processes including subvocal rehearsal seem to be at place around age 7 [45–46]. Thus, testing children around the onset of their full working memory capacity tests the boundary limits of the hypothesized relationship and also its developmental perspective. This study also contributes to exploration of perceptual abilities correlated with working memory in children close to or within the critical period for the first language acquisition, as related to brain plasticity and age (cf. [47]).

## Experiment

We asked whether young children's performance on a prosodic discrimination task correlates with standard working memory measures. We used a same-different discrimination task for the participants to differentiate between pairs of phonemically identical but prosodically different pairs of syntactically well-formed sentences. In addition, participants were tested on: i) forward digit span (FDS); ii) backward digit span (BDS) and iii) non-word repetition (NWR). The FDS and NWR tasks are commonly used to test the storage component of the working memory [44,48]. The BDS task imposes a substantial processing load on the participant, hence is considered to involve the processing component of the working memory as well [49]. As previous research revealed a non-trivial role of bilingualism in prosodic discrimination task, a complementary goal of the present study was to replicate the bilingualism effect in the prosodic discrimination ability reported in [22].

## Method

### Participants

Seventy Slovenian-speaking monolingual [N = 35, M_age_ = 9.17, SD = 0.39] and closely age-matched bilingual [N = 35, M_age_ = 9.15, SD = 0.34; *t*(68) = 0.16, *p* = 0.87] children from elementary schools in Gorizia and Nova Gorica, two towns on the respective sides of the Italian-Slovenian border, were tested. Children's bilingual status was determined on the basis of a parent questionnaire. Seventeen (49%) of the bilingual children were exposed to both languages from birth, fifteen others (42%) by age 2, and three were exposed to the second language between their 3 and 6 years. One of the languages spoken by the bilinguals was always Slovenian; the other language was predominantly Italian, or, alternatively, Bosnian/Croatian/Serbian, English, Friulian, German, Macedonian, Portuguese, Russian or Spanish. None of the tested participants had a previous exposure to French. Three and seven monolingual children (8% and 20%) had up one and two years of systematic musical instrument training, respectively. The participants had normal or corrected to normal vision and no history of hearing disorders. Two children from the tested pool had a minor articulatory deficiency (mispronouncing the "r" sound), which was taken into account when decoding their results from the NWR task. Another two children's (one monolingual, one bilingual) results from the discrimination task were not included in the correlation analyses: one child's data was lost due to an experimenter error; the other's because of a near chance performance on phonemically different controls (a 50% accuracy threshold was assumed). The experimental protocol was approved by the Ethics Committee of the University of Nova Gorica (ref. no. 24-1/2017) and was carried out in accordance with the relevant guidelines and regulations. Informed consent was obtained from the participants’ legal guardian/s.

### Design and materials

#### Discrimination task

**Fig 1:**
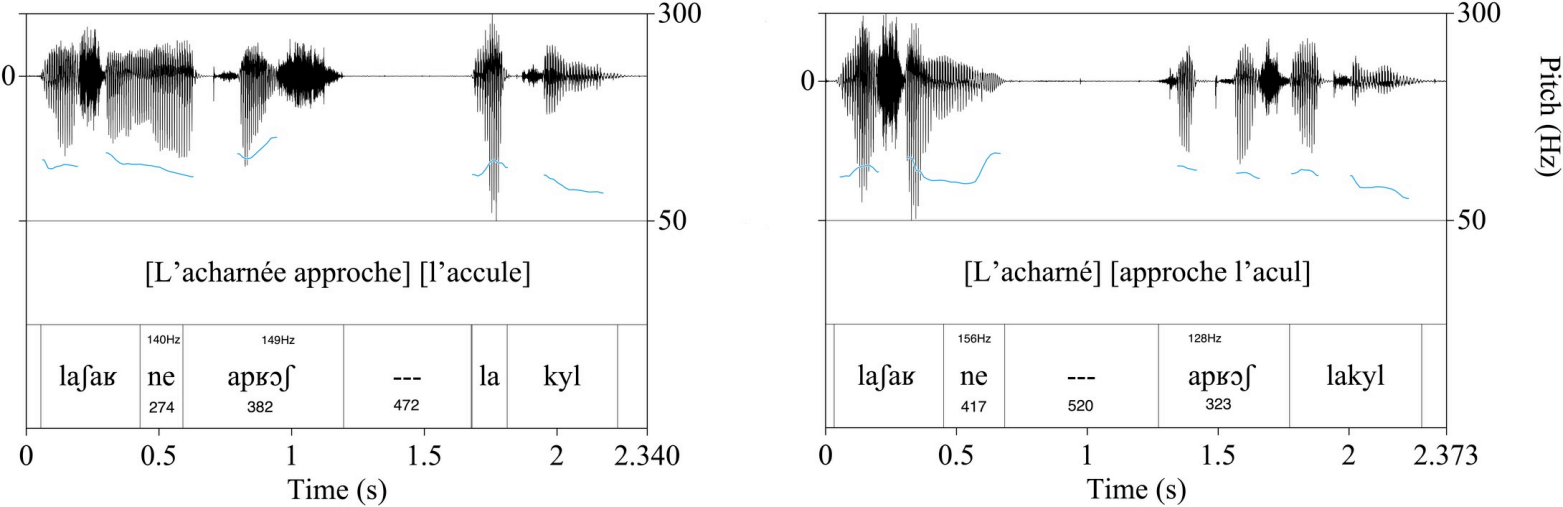
**Prosodic features of the noun condition (on the left) and verb condition (on the right) in the sentence discrimination task.** Segmental durations and maximal F0s in critical regions are shown underneath and above the IPA notation, respectively.

The stimuli were a subset of materials used in [22] and included pairs of short sentences in French, the language unknown to participants and notably distinct in its prosodic properties from the participants' native language, Slovenian (e.g. in the lack of lexical stress). Sentence pairs were constructed in such a way so as to exploit the high degree of cross-categorical lexical homonymity in French: words that differ in syntactic category, e.g. noun and verb, can have the same phonological and/or phonetic makeup, e.g., *ferme* for “farm” or “closes.” Specifically, the following cross-categorical ambiguities were exploited: a) between nouns and verbs, b) between nouns and adjectives and c) between definite determiners and phonemically identical weak pronouns or clitics (*le*, *la*, *les*). To illustrate, the sentences [_NP_ *Le jeune garde*][_VP_ *la voit*] "The young guard sees her" and [_NP_ *Le jeune*][_VP_ *garde la voie*] "The young man guards the road" are lexically and syntactically different, but sound very similar because there are little or no phonological (segmental or word-level) difference. The only potentially detectable difference is prosodic (supra-segmental): each sentence is pronounced with a different prosodic contour involving a constellation of prosodic cues (see below). As the bracketing in the above pairs of examples indicates, there are two prosodic groupings in each sentence corresponding to respective syntactic constituents: a noun phrase (NP) and a verbal phrase (VP) separated by a prosodic boundary. In one condition, termed noun condition, a long NP is followed by a short VP (the noun condition), in the other condition, termed verb condition, a short NP is followed by a long VP (the verb condition). Prosodic cues that are likely to influence and facilitate discrimination in a given pair of target sentences include: i) phrase-final segment lengthening at the prosodic/syntactic boundary between NP and VP [50]; ii) phrase-initial articulatory strengthening, whereby the onset of the first post-boundary syllable (e.g., /g/ in *garde* in the above examples) is lengthened in the verb condition compared to the noun condition [51]; iii) a silent pause between the prosodic NP and VP units; iv) a pitch rise whenever a (phrase-final) word precedes a prosodic boundary, as opposed to the condition in which it does not (e.g. *jeune* in the noun condition compared to *jeune* in the verb condition above), consistently with the general pattern of the rising pitch contour toward the end of prosodic units in French. These boundary cues are typical for French, but in other combinations may also occur in other languages, suggesting that children's sensitivity in this case may be a function of general acoustic salience [1,2,52]. The prosodic signatures of a sample target sentence pair are illustrated in Fig 1.

We used 16 phonemically identical-prosodically different stimulus pairs as target pairs, 16 pairs of phonemically and prosodically different sentences, 16 pairs of phonemically and prosodically identical sentences serving as controls, and 16 more pairs of identical filler sentence pairs, to ensure that “different” answers do not predominate creating a habituation among subjects (note that the suprasegmental nature of prosody which feeds on the segmental component makes it highly non-trivial to utilize a logically possible condition with phonemically different but prosodically identical sequences; we therefore used a simpler design manipulating the prosodic factor over the same phonemic units).

Acoustic analyses of duration and pitch on the phonemic segments in the area surrounding the prosodic boundary were conducted (see Fig 1). Total duration of sentences in each condition did not differ significantly (*M*_noun_ = 2248ms, *SD*_verb_ = 32 vs. *M*_verb_ = 2263ms, *SD*_verb_ = 20; *t*(30) = 0.25, *p* > 0.10). The time until the onset of the prosodic boundary demarcating the noun and verb phrases differed across the conditions (*M*_noun_ = 1129ms, *SD*_verb_ = 157 vs. *M*_verb_ = 684ms, *SD*_verb_ = 136; *t*(30) = 8.57, *p <* 0.001). The segment preceding the prosodic phrase boundary in the noun condition (e.g *approche* in Fig 1) was lengthened by 18% compared to the verb condition (*M*_noun_ = 382ms, *SD*_verb_ = 97 vs. *M*_verb_ = 323ms, *SD*_verb_ = 98; *t*(30) = 1.82, *p* = 0.03). The segment immediately preceding the prosodic phrase boundary in the verb condition (e.g. *ne*) was lengthened by 52% compared to the noun condition (*M*_verb_ = 417ms, *SD*_verb_ = 104 vs. *M*_noun_ = 274ms, *SD*_verb_ = 81; *t*(30) = 6.41, *p <* 0.001). These observed patterns are consistent with the previous literature on French intonation [50]. The prosodic boundaries in our stimuli are also marked by a (systematically present) silent pause between the noun and verb phrases, whose duration was comparable across both conditions (*M*_noun_ = 472ms, *SD*_noun_ = 162 vs. *M*_verb_ = 520ms, *SD*_verb_ = 124; *t*(30) = 0.92, *p* > 0.10).

For each pair of stimulus sentences, we also compared maximum F0s on the words to be found on different sides of a prosodic boundary in the verb condition, but on the same side in the noun condition, e.g. *l’acharnée* and *approche* in Fig 1, henceforth referred to as Word 1 and Word 2. This analysis revealed a significant rise of pitch when the word immediately precedes a prosodic boundary (Word 1: F0^max^ (noun condition) = 140Hz, F0^max^ (verb condition) = 156Hz, *t*(30) = 1.73, *p* = 0.04; Word 2: F0^max^ (noun condition) = 149Hz, F0^max^ (verb condition) = 128Hz, *t*(30) = 3.12, *p* = 0.001). The average percentage of rise between the F0 minima and F0 maxima located on Word 1 is greater in the verb condition (83.15%) than in the noun condition (40.68%). These patterns are consistent with the general tendency of French for a rising pitch contour towards the end of prosodic units.

Overall there were 64 stimulus pairs. Within target pairs, the order of presentation between the two syntactic structures was counterbalanced, with 8 stimuli beginning with the noun condition, and 8 with the verb condition. The sentences were also balanced by length which amounted to 7 ± 2 syllables. The stimuli were pre-recorded by a male native French speaker with an interval of 5 seconds between the sentences within each pair. Speech was digitized to a computer with a sampling rate of 44.1 KHz and a 16-bit sampling depth, onto two stereo channels.

#### Digit span tasks

We used the visual, rather than verbal, version of the tasks in order to avoid potential confounds connected with participants' bilingualism and dominant language. It was assumed that the children were able to count to 10, in line with their current level of school education. In the FDS, children were asked to repeat sequences of digits in the same order, whereas in the BDS, they repeated them in reverse order.

#### Non-word repetition task

This task was modeled on Children's test of Nonword repetition (CNRep, [48]) which we adapted for the Slovenian language. Forty non-words were constructed while carefully controlling for the Slovenian phonotactic restrictions including restrictions on consonant clusters within a syllable and on stress patterns for words of similar length and syllable complexity, as well as a reasonably balanced distribution of the standard language's vocalic repertoire [53–55]. Only phonotactically admissible CVC, CCVC, and CVCC consonantal clusters within a syllable were used. Stress was placed in locations typically occurring in actual Slovenian words with a similar syllable structure. This way we ensured that segment (phoneme) sequences within each non-word were phonotactically and prosodically legal. For consistency, all non-words began with a consonant. The length of non-words varied between 2–5 syllables long and was balanced across the stimuli (10 nonwords of each length). The stimuli were pre-recorded by a female native speaker of Slovenian and digitized into .wav files using Praat [56].

### Procedure

Children were tested in a quiet room on school premises. The experiment was administered in two separate sessions, with an interval of 1–3 days between the sessions for each child. One session included the prosodic discrimination task, the other included the FDS, BDS and NWR tasks. Approximately half of the children were tested first on the discrimination task followed by the working memory tasks, whereas the other half were tested in the reversed order. Each session lasted between 15–20 minutes in total. For their contribution, the participants were rewarded a pen or a sticker.

#### The discrimination task

Participants heard pairs of stimulus sentences played binaurally at a comfortable listening level and were asked to determine whether these sentences sound the same or different. Stimuli were presented in a pseudo-randomized order different for each child, with no prior familiarization stage. There was a self-timed break after 32 stimulus pairs. During a practice trial, participants listened to six exemplars of each experimental condition through computer-internal loudspeakers and were given feedback by the experimenter.

#### The digit span tasks

Digits in each sequence appeared on the screen for 1000 milliseconds one after another with no inter-stimulus interval. There were four trials per block. Each task started with sequences of three (randomly chosen) digits, and got progressively longer by one. When the child repeated the first three trials within a block of four correctly, the task automatically continued with the next block. In case of non-contiguous succession of correct answers, all four sequences were presented. The task began with few practice trials followed by the main experiment. The experiment stopped after two or more (≥50%) incorrect trials within one block.

#### The non-word repetition task

Non-words were played back to participants via the headphones at a comfortable listening level, with a time interval of 5 seconds between the stimuli, during which the participant had to repeat the stimulus. Stimuli were presented to the participants in the pseudo-randomized order. Participants' answers to each stimulus were recorded for later analysis.

## Results

### The discrimination task

**Fig 2:**
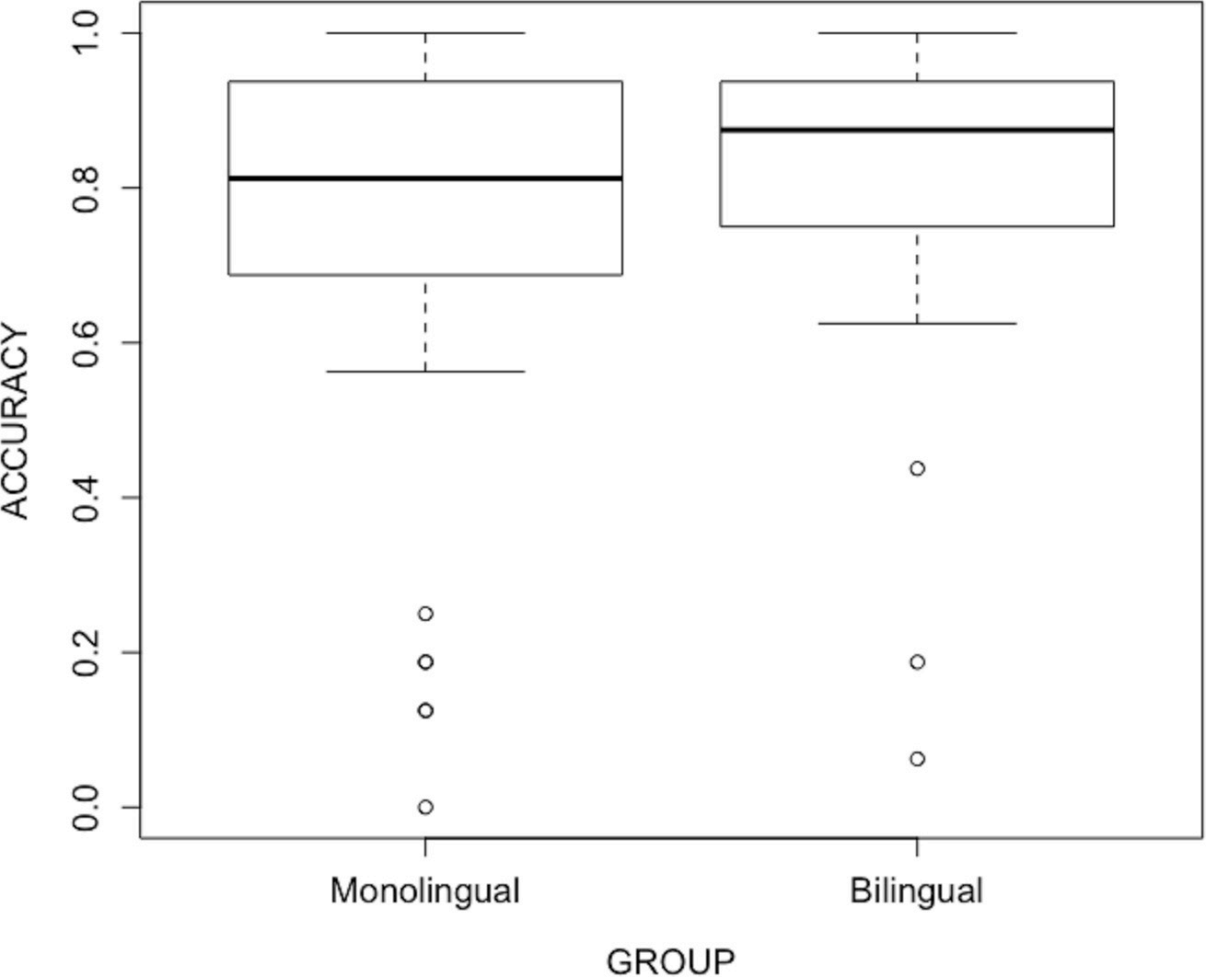
Accuracy score means per participant on target trials, across the bilingualism factor.

Response biases were controlled by calculating hit and false alarm rates and the *d*′ value for each participant over all conditions. *D*′ values were significantly larger than 1.35 [*t*(69) = 10.59, *p* < 0.0001], indicating an overall accuracy of more than 75% [57]. Accuracy on phonemically different pairs was 97.1% (95.6% for monolinguals, 98.7% for bilinguals), and overall mean response time (RT) was 1479 msec (SD = 683). Within the phonemically identical pairs, mean accuracy was 89.6% (88.6% for monolinguals, 90.6% for bilinguals) on prosodically identical control pairs, and 77.0% (71.7% for monolinguals, 82.3% for bilinguals) on prosodically different target pairs. RTs over the entire participant pool were 1403 msec (SD = 684) and 1385 msec (SD = 672). Bilingual participants showed better performance than monolingual ones in terms of number of correct discriminations (chi-square test: χ^2^(1) = 11.412, *p* = 0.0007). Distribution of accuracy scores on phonemically identical-prosodically different trials per participant across the bilingualism factor is shown in Fig 2.

### The digit span tasks

A scoring procedure was applied based on the number of correctly scored sequences (cf. [58]). A trial was scored with 1 point if a sequence was recalled correctly, and with 0 points if participants recalled one or more digits in a sequence incorrectly or if they omitted one or more digits. Thus, for instance, if the task stopped at the level of 5-digit sequence, a participant's score could vary between 6 and 8 (3 at the 3-digit level + 3 at the 4-digit level + 0–2 at the 5-digit level).

Scores on the FDS sequences (maximum length attained = 8) varied between 2 and 15, with a total average of 8.61 (SD = 2.47). Scores on the BDS sequences (maximum length attained = 6) varied between 4 and 11, with a total average of 6.17 (SD = 1.68). There was no significant difference between monolinguals and bilinguals in the performance on the FDS (M_mono_ = 9.14, M_bi_ = 8.08, t(63.7) = 1.79, *p* = 0.077) and BDS (M_mono_ = 6, M_bi_ = 6.35, t(63.6) = 0.86, *p* = 0.39).

### Non-word repetition task

Participants' answers in the recordings were manually coded for correctness of repetition by a native Slovenian speaker (one of the experimenters) who was aware of the task. A random 10% of the data were additionally coded by another Slovenian speaker who was not aware of the purpose of the task. Agreement between the raters was 94.3% and Cohen's unweighted kappa was 0.88 indicating a high degree of inter-rater reliability.

**Fig 3:**
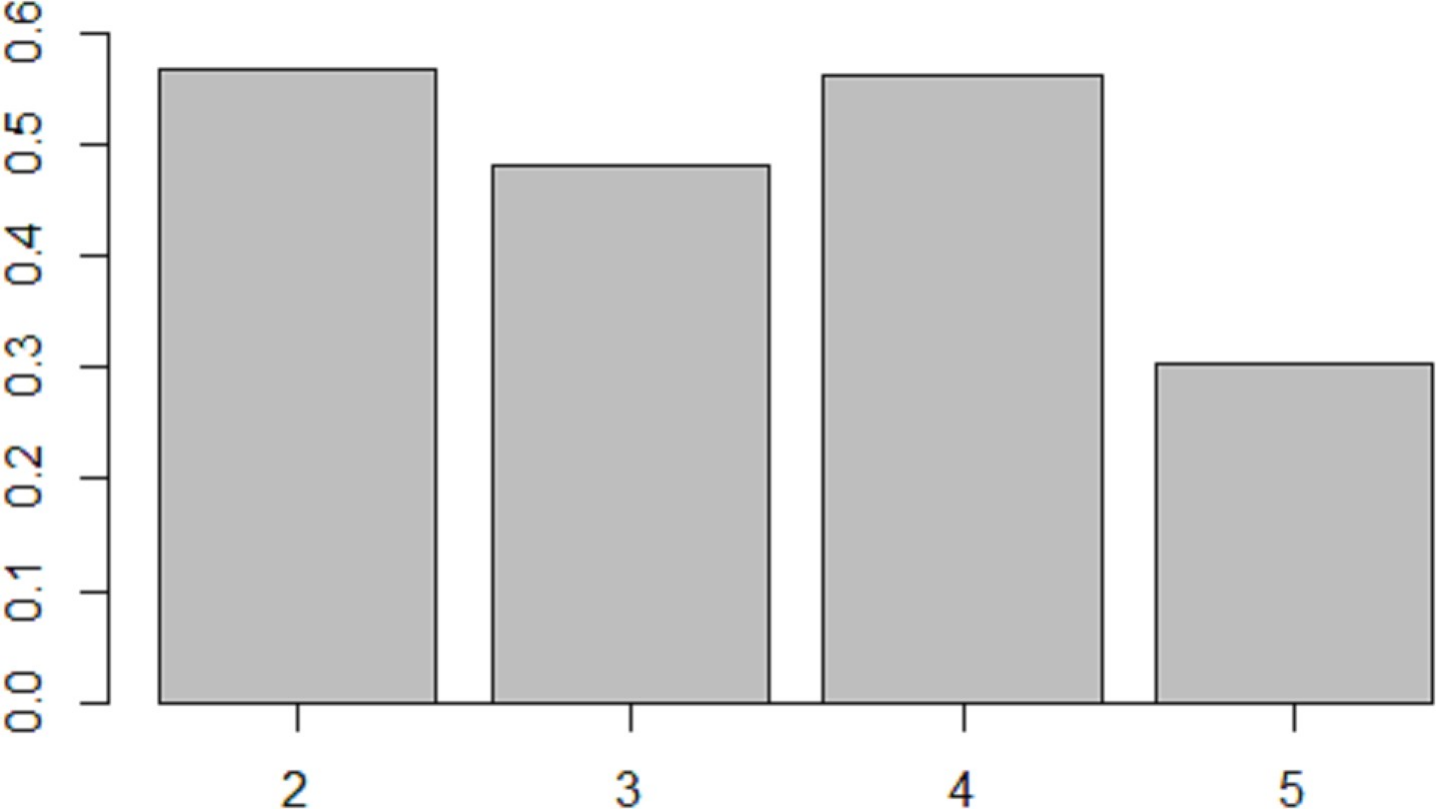
Overall accuracy in the non-word repetition task as a function of number of syllables.

**Fig 4:**
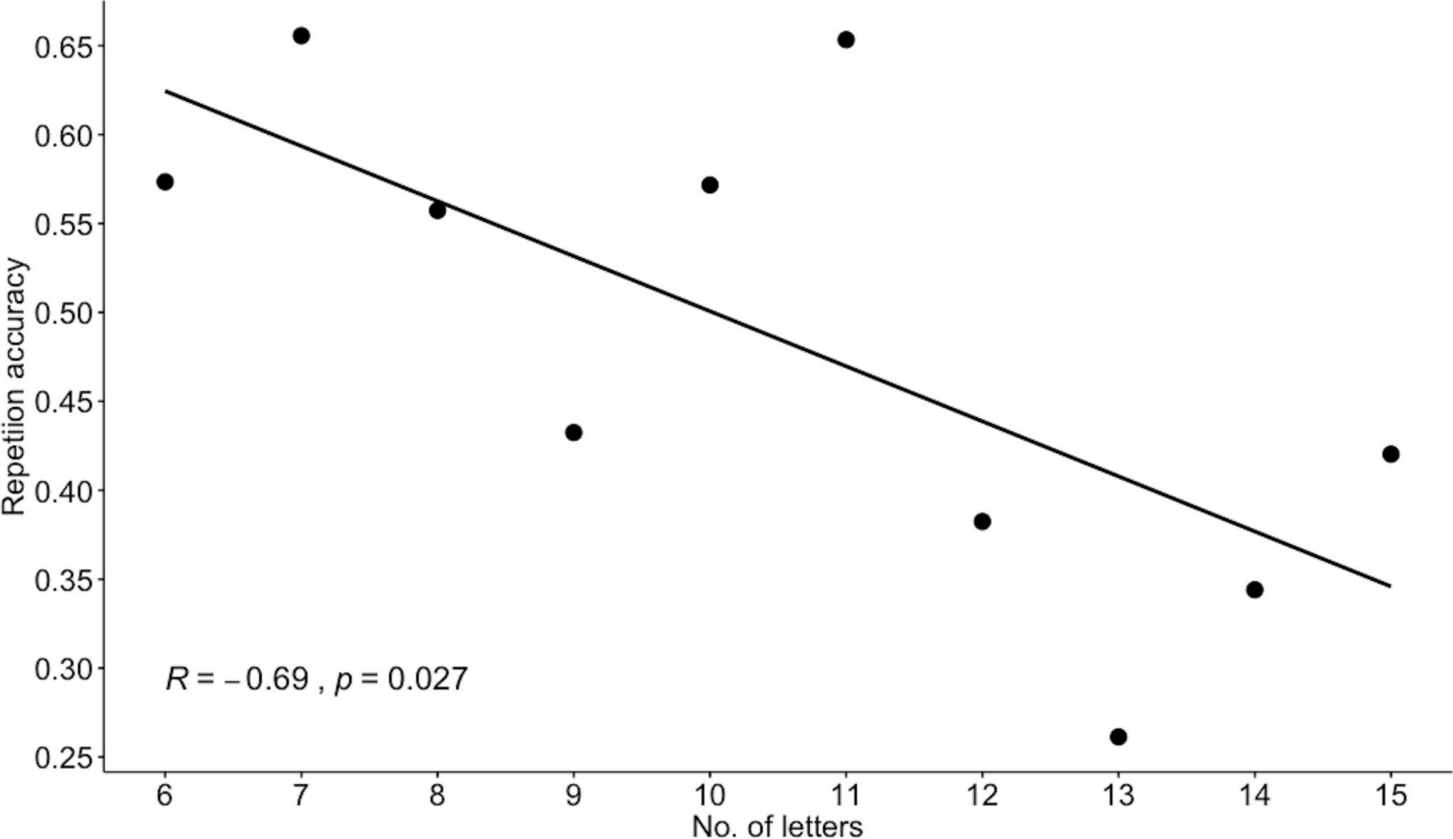
The word length effect in the non-word repetition task as a significant correlation between overall accuracy and non-word length in letters.

The overall success on all items was 48% (*SD* = 15). Types of repetition errors that lead to registering a response as incorrect included: i) consonantal and vocalic omissions; ii) assimilation to a preceding or following consonant; iii) metathesis; iv) consonantal and vocalic insertions; v) substitutions; vi) incomplete productions. The overall accuracy across the four syllable lengths is summarized in Fig 3. Bilingual and monolingual participants did not differ in their overall repetition accuracy (*t*(59) = 0.70, *p* = 0.48). Pearson's product-moment coefficient indicated a significant negative correlation between repetition accuracy and the length of non-words, as shown in Fig 4. The latter finding corroborates the results reported in previous studies of non-word repetition in various types of populations and is consistent with the models of working memory that involve a p-STM component with a rehearsal procedure, (cf. [59–60]).

### Correlation analyses and models

**Fig 5:**
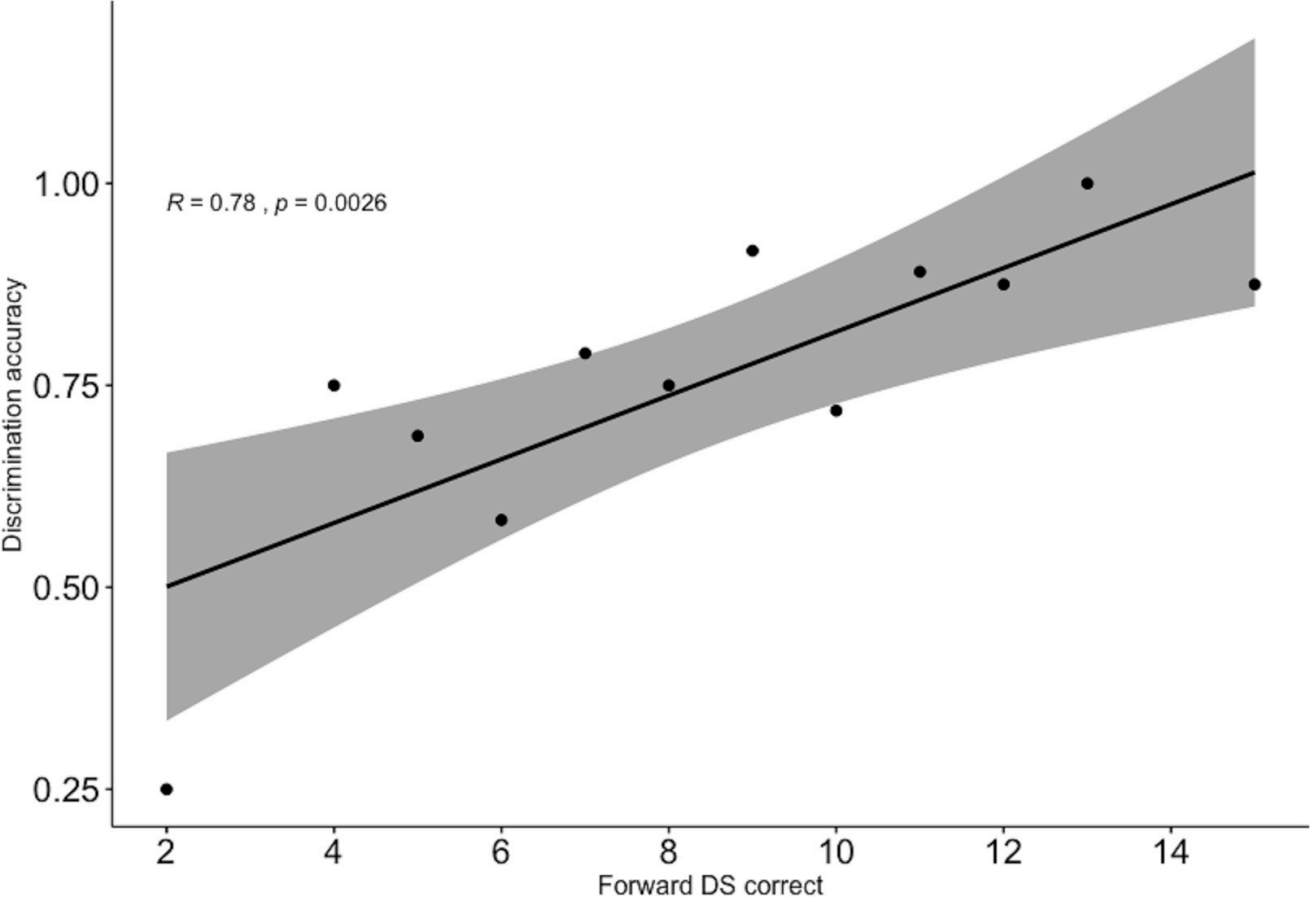
Correlation between discrimination accuracy and forward digit span task (aggregated participant results). Confidence interval is shown in grey.

**Fig 6:**
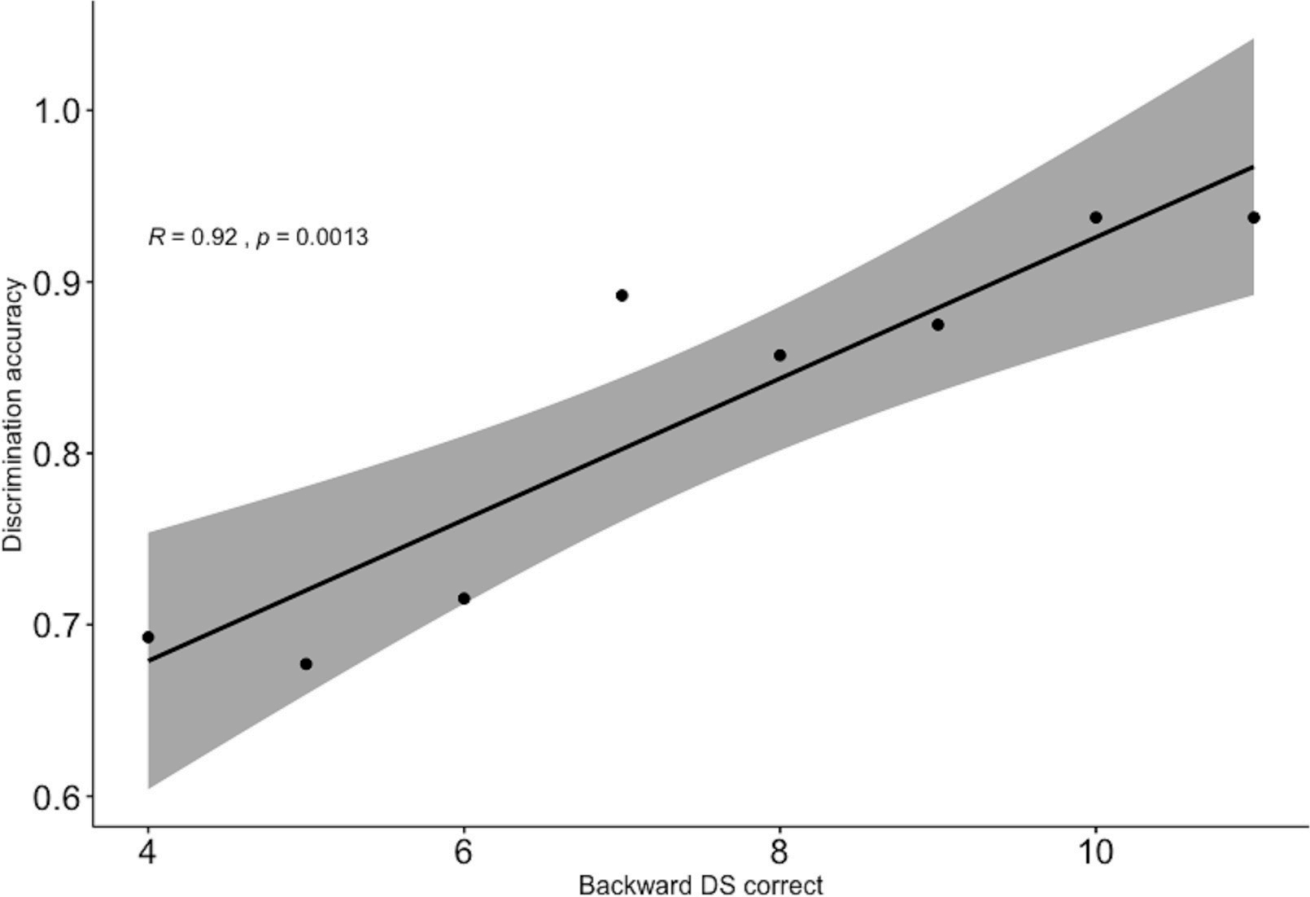
Correlation between discrimination accuracy and backward digit span task (aggregated participant results). Confidence interval is shown in grey.

**Fig 7:**
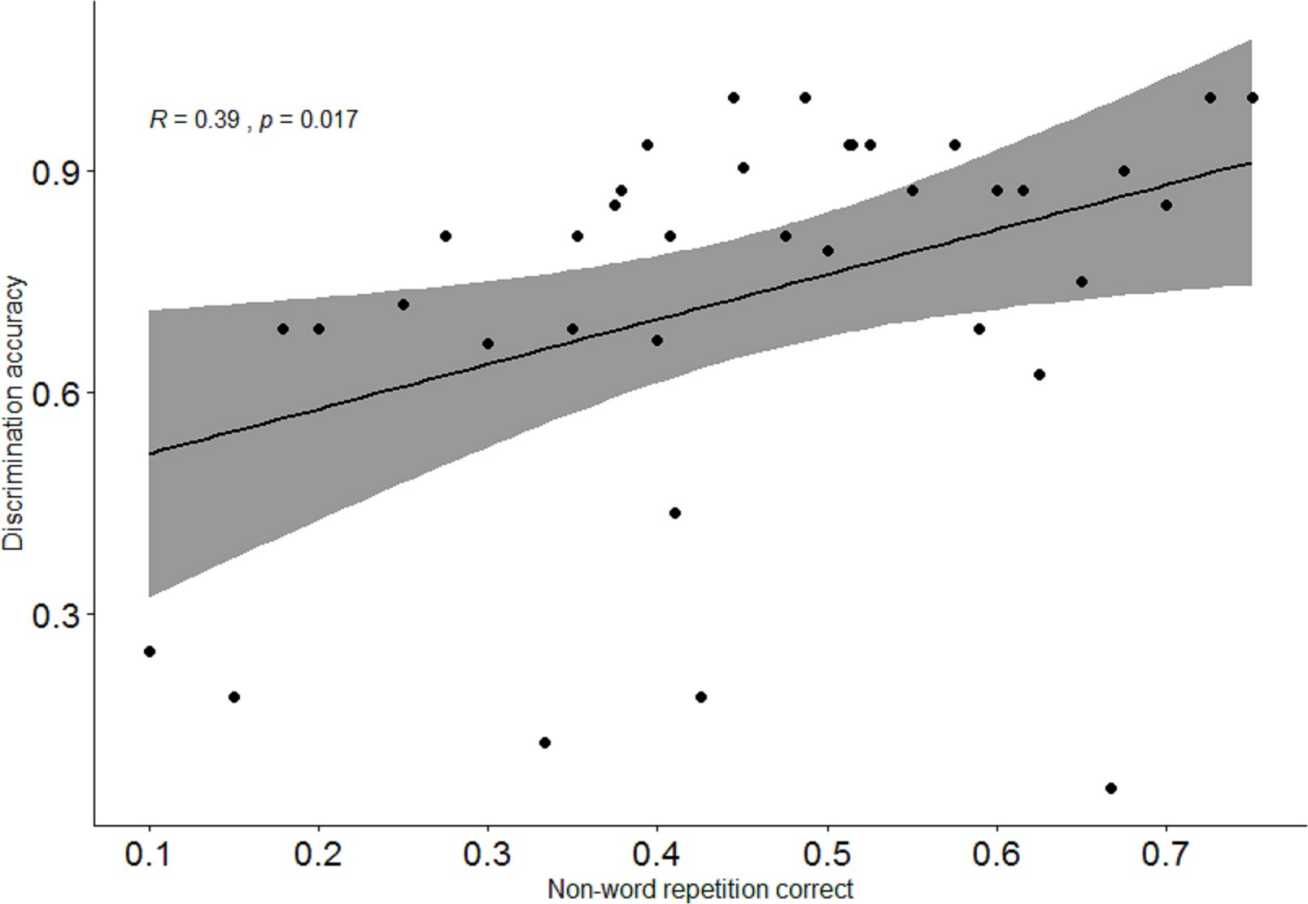
Correlations between discrimination accuracy and non-word repetition task (aggregated participant results). Confidence interval is shown in grey.

Focusing on the subset of monolingual and bilingual children’s responses on the target (phonemically same, prosodically different) pairs, significant positive correlations were observed between the success ratios in the discrimination task and each of the three other measures. In addition, correlations were also observed between the non-word repetition and each of the FDS and the BDS tasks, as well as between the FDS and BDS tasks themselves. Table 1 and Figs 5–7 show correlations in the raw and aggregated participant data, respectively (aggregation is performed by taking the mean over accuracy in discrimination task and success in each of working memory measures).

**Table 1:** Correlations between accuracy scores in the discrimination and the WM tasks, raw (non-aggregated) participant data.

	Discrimination	FDS	BDS
**FDS**	0.31*		
**BDS**	0.32*	0.35*	
**Non-words**	0.32*	0.51***	0.29*

* < 0.05

*** < 0.001

Since performance on each of the three WM tasks correlated with discrimination accuracy, we were also interested to know to what extent it predicts individual same-different responses in the discrimination task. To that end, mixed effects logistic regression models were constructed using the *lme4* package in R version 3.5.3 [61–62], with accuracy on the discrimination task as the (categorical) binary dependent variable. Participant and item were treated as random factors with intercepts, with bilingualism status as a random slope. We used a stepwise upward model selection procedure whereby fixed factors were progressively added in such a way that the Akaike Information Criterion (AIC) was minimized indicating better fit. Three of the four fixed factors of interest, bilingualism, performance on the FDS and BDS tasks improved the model fit and were thus added in the final model. The NWR performance did not improve (in effect, worsened) the model fit and therefore was not added. The results of the model with the three fixed factors are reported in the upper portion of Table 2. Further explorations of the model revealed that the NWR performance improves the model fit just in case the FDS factor was omitted. The outcome of the so adjusted model is reported in the lower portion of Table 2 (estimates are provided in logarithms of odds of giving a targeted answer, converted to probabilities by taking an inverse logit: [logit^−1^(α) = exp(α)/(1 + exp(α)]).

**Table 2:** Model estimates predicting accuracy in discrimination task using bilingualism and WM measures as fixed factors.

*Fixed factor*	*β*	logit^−1^(*β*)	*SE*	*z*	Pr(>|*z*|)
(Intercept)	-1.7420	0.1490	0.9198	-1.894	0.0582.
BILING	0.6482	0.6566	0.3114	2.082	0.0374*
FDS	0.2132	0.5531	0.0907	2.351	0.0187*
BDS	0.2718	0.5675	0.1337	2.033	0.0421*
BILING × FDS	-0.1931	0.4518	0.1155	-1.671	0.0946.
BILING × BDS	-0.0088	0.4978	0.1737	-0.051	0.9596
FDS × BDS	0.0136	0.5034	0.0512	0.266	0.7899
NWR	2.7935	0.9423	1.3816	2.02[1]2	0.0432*
NWR × BILING	-3.0267	0.0462	1.9771	-1.531	0.1258
NWR × BDS	0.2621	0.5651	0.8185	0.320	0.7490

. < 0.1

* < 0.05

As expected, the models revealed main effects of the bilingualism factor as well as performance in the FDS and BDS tasks, as well as the NWR task, with each of the corresponding estimates signaling an increase in respective probability of correct discrimination by degree indicated in the third column in Table 2. There was, however, no significant two-way interactions between the predictors themselves (respective three-way models did not converge), with the only interaction Bilingualism*FDS improving the model fit somewhat. We also run linear mixed effects models on participants' RTs using the *lme4* package for R (only RTs on the correct responses were analyzed). RTs were similar across the conditions and no significant predictors emerged in this case (all *p*s > 0.10).

## Discussion

We found that children's performance on the relevant working memory measures positively correlated with their overall performance on the discrimination task and also predicted individual discrimination decisions. These results supported the hypothesis that both the storage and processing components of the working memory are at play. However, we observed no interaction of the respective working memory measures, suggesting that a greater storage as well as processing capacity do not necessarily lead to a better success in prosodic discrimination performance than each of these components on its own.

Another important result of the present study is a replication of the bilingualism effect in prosodic discrimination [22]. The extent to which bilinguals enjoy cognitive advantages at different ages is currently under debate in the literature [43, 63]. The present study supports the idea that in the domain of prosodic differentiation at the sentence level, bilingual elementary school-aged children enjoy a stable perceptual advantage, which is also consistent with studies reporting bilinguals' better success at foreign language learning (e.g. [13]). It is important to note, however, that this advantage cannot be directly associated with better working memory in bilinguals, in particular, the p-STM and/or processing component, as indicated by the absence of bilingualism effects in our administered WM measures and weak to no interactions between performance markers in the discrimination task and the WM tasks. This should not appear surprising given that the previously observed cognitive advantages related to bilingualism pertain mostly to executive control, to the extent that the latter is distinct from and/or covers different functions than p-STM (cf. Introduction).

A further question is the extent to which (specific components of the) working memory are deployed in the prosodic discrimination task. A pertinent line of inquiry explored in the literature concerns the chunking process. It has been suggested that listeners do not interpret speech on a sound-by-sound basis, but base their decisions over some perceptual unit that can span a number of elements [64]. Given the limited capacity of p-STM, it has been suggested that a chunking process applies in speech processing creating smaller chunks of input in the working memory [23, 28, 65]. These chunk-like units are detectable, for instance, by specific ERP components in speech processing [10, 66–70]. A better p-STM capacity would thus be associated with a greater number of stored prosodic units, or, given a limited number of those (as is the case in the present study), their stronger memory traces, as well as a better segmentation process.

Broadly speaking, comparing two sentence-level prosodic signatures in the present study is also similar to comparison of two short musical melodies. Brain imaging studies using different methodologies consistently indicate that, in melody discrimination tasks, brain structures associated with auditory working memory are activated to a greater extent in musically untrained children than in musically trained ones, likely due to less auditory experience with music sampling and sequencing [71–73]. Consequently, a greater working memory capacity is again beneficial in this regard.

Other cognitive skills that we have not controlled for in the present study could be additional predictors of the prosodic discrimination performance. One potential candidate is phonological and especially phonemic awareness, the ability to consciously recognize and manipulate specific sounds and sound combinations in an auditory input in predictable ways [74]. In addition, phonological awareness, on the one hand, and p-STM or phonological loop, on the other, may have a common cognitive underpinning [75]. Further research should explore the role of this and other related factors in better understanding the mechanisms behind prosodic discrimination.

The results of the present study are also consistent with the developmental models according to which the full working memory capacity responsible for adult-like performance on the working memory/p-STM tasks is by and large available at age 9 and later (see the references in Introduction). A further interesting question would be how the prosodic sensitivity at the sentence level is affected in various pathological circumstances (e.g. brain damage, hearing loss etc.) in the developmental context, alongside potential collateral effects on the working memory. This, together with other developmental factors that may affect performance in the sentence-level prosodic discrimination task, is among possible directions for further inquiry.
